# Relationship between periodontitis and atrial fibrillation recurrence after ablation: a systematic review

**DOI:** 10.3389/fcvm.2026.1764472

**Published:** 2026-02-10

**Authors:** Xinnian Luo, Chuansong Zou, Yong Liu

**Affiliations:** 1 Department of Stomatology, Ganzhou People’s Hospital, Ganzhou, Jiangxi, China; 2 Department of Stomatology, Chongyi County Ankang Traditional Chinese Medicine Hospital, Ganzhou, Jiangxi, China; 3 Department of Stomatology, Shicheng County People’s Hospital, Ganzhou, Jiangxi, China

**Keywords:** atrial fibrillation, catheter ablation, periodontitis, recurrence, systemic inflammation

## Abstract

**Background:**

Atrial fibrillation (AF) recurrence after catheter ablation remains a significant clinical challenge, with chronic inflammation implicated in its pathogenesis. Periodontitis, a prevalent chronic inflammatory disease, may contribute to AF recurrence through systemic inflammation and microbial translocation. This systematic review synthesizes evidence on the association between periodontitis and AF recurrence post-ablation.

**Methods:**

MEDLINE, EMBASE, and Cochrane Library were searched from inception to January 2026. Observational studies evaluating periodontitis (diagnosed clinically, serologically, or via periodontal indices) and post-ablation AF recurrence in adults were included. Study quality was assessed using the Newcastle-Ottawa Scale. Narrative synthesis was performed due to methodological heterogeneity.

**Results:**

Three observational studies (*n* = 1,016 patients) met inclusion criteria. Periodontitis prevalence ranged from 54% to 57%. All studies reported significant associations between periodontitis and increased AF recurrence risk, including elevated Porphyromonas gingivalis antibodies [paroxysmal AF: hazard ratio (HR) = 1.57; 95% confidence interval (CI), 1.01–2.43; non-paroxysmal AF: HR = 1.91; 95% CI, 1.21–3.01], clinical periodontitis (HR = 2.06, 95% CI, 1.02–4.18), and high Periodontal Inflamed Surface Area (PISA >615 mm^2^; HR = 2.31, 95% CI, 1.23–4.32). Mechanistically, systemic inflammation, reflected by elevated C-reactive protein (CRP), interleukin-6 (IL-6), and tumor necrosis factor-α (TNF-α), and pathogen-specific atrial remodeling were implicated.

**Conclusion:**

Periodontitis is consistently associated with higher AF recurrence after ablation, likely mediated by systemic inflammation and oral pathogen activity, highlighting periodontitis as a modifiable risk factor for AF recurrence.

## Introduction

Atrial fibrillation (AF), the most prevalent sustained cardiac arrhythmia, constitutes a major global health burden due to its strong association with stroke and cardiovascular morbidity (1–3). Catheter ablation has emerged as a cornerstone therapy for rhythm control, yet post-procedural AF recurrence remains common, reflecting the complex interplay of structural remodeling, systemic inflammation, and other poorly understood mechanisms (4, 5).

Chronic low-grade inflammation is increasingly recognized as a key contributor to AF pathogenesis and progression (6–8). This inflammatory milieu may originate from diverse sources, including, for example, metabolic disorders, obesity, and notably, periodontitis (a chronic inflammatory disease affecting the tooth-supporting tissues). Periodontitis, which affects nearly half of the adult population worldwide, is associated with a systemic inflammatory state reflected by elevated circulating markers such as C-reactive protein (CRP), interleukin-6 (IL-6), and tumor necrosis factor-α (TNF-α) (9, 10). While these biomarkers primarily serve as indicators of systemic inflammation rather than disease-specific mediators, epidemiological and mechanistic studies suggest that chronic periodontal inflammation may contribute to atrial remodeling and increased susceptibility to AF (11, 12).

Recent clinical investigations have extended this paradigm to AF ablation outcomes. Despite these advances, the evidence base remains limited and methodologically heterogeneous. Existing studies employ divergent periodontitis assessments, ranging from serologic markers to clinical periodontal exams, and vary in patient populations (paroxysmal vs. persistent AF), follow-up durations, and outcome definitions (13–15). In view of these gaps and mixed results, a systematic review is warranted to synthesize current data on periodontitis and AF recurrence after ablation, clarify the strength of the association, and guide future investigation.

## Methods

As this is a systematic review analyzing previously published studies, ethical approval is not required. The data supporting the findings of this study are derived from publicly available sources, all of which are cited within the manuscript.

### Literature search strategy

A systematic literature search was conducted. Electronic databases, including MEDLINE (via PubMed), EMBASE, and the Cochrane Library, were systematically queried from inception to January 2026 using a combination of Medical Subject Headings (MeSH) and free-text keywords. Terms related to periodontitis (e.g., “*periodontal disease*”, “*periodontitis*”, “*gingivitis*”, “*oral inflammation*”) were combined with terms pertaining to atrial fibrillation recurrence (e.g., “*atrial fibrillation recurrence*”, “*AF ablation failure*”, “*post-ablation AF*”, “*arrhythmia recurrence*”) using Boolean operators (AND/OR). No language restrictions were applied. To ensure comprehensiveness, additional studies were identified through manual screening of reference lists from included articles and relevant reviews.

### Study selection criteria

Eligibility criteria were defined according to the PICOS framework. The population comprised adults (≥18 years) undergoing catheter ablation for AF. The exposure of interest was periodontitis, diagnosed through clinical criteria (e.g., probing depth, clinical attachment loss, and bleeding on probing), radiographic evidence, or validated indices such as the periodontal inflamed surface area (PISA). The comparator consisted of patients without periodontitis. The primary outcome was documented AF recurrence following ablation, confirmed by electrocardiogram (ECG), ambulatory monitoring, or implantable devices after the blanking period. Observational studies evaluating periodontal status or periodontal treatment (non-randomized) in relation to AF recurrence were eligible.

Exclusion criteria encompassed animal studies, reviews, editorials, conference abstracts, cross-sectional studies, and studies lacking explicit data on AF recurrence or periodontal status. Randomized controlled trials were not identified in this domain and thus were not included.

### Study screening and data extraction

Two independent reviewers (X.L. and C.Z.) screened titles and abstracts, followed by full-text assessments for eligibility. Disagreements were resolved by consensus. A standardized data extraction form was used to collect key study characteristics (author, year, country, design), patient demographics (sample size, age, comorbidities), periodontal assessment methods, AF recurrence ascertainment techniques, and adjusted effect estimates [hazard ratios (HRs) or odds ratios (ORs)] with 95% confidence intervals (CIs), including covariates controlled for in multivariate models.

### Quality assessment

The methodological quality of included cohort and case-control studies was evaluated using the Newcastle-Ottawa Scale (NOS), which assesses three core domains: selection (e.g., representativeness, exposure ascertainment), comparability (adjustment for confounders such as age, sex, hypertension, diabetes), and outcome (AF recurrence assessment, adequacy of follow-up). Studies scoring ≥6 were classified as moderate-to-high quality (16–18). Risk of bias was assessed independently by two reviewers (X.L. and C.Z.), and discrepancies were resolved by consensus.

### Data synthesis

Due to anticipated heterogeneity in periodontitis definitions (e.g., clinical vs. serologic vs. PISA index) and AF recurrence monitoring protocols (e.g., variable follow-up intervals, ablation techniques), a meta-analysis was not conducted. Instead, a narrative synthesis was performed, focusing on the direction, consistency, and magnitude of associations across studies, along with study quality and mechanistic insights (e.g., systemic inflammation, microbial translocation).

Although the protocol was not registered, the review question, eligibility criteria, and analysis plan were predefined before literature screening to minimize selective reporting bias. This systematic review was conducted and reported in accordance with the Preferred Reporting Items for Systematic Reviews and Meta-Analyses (PRISMA) 2020 statement (19). A completed PRISMA 2020 checklist, indicating the location of each reporting item within the manuscript, is provided as Table 1.

**Table 1:** Baseline characteristics of included studies.

Included studies	Miyauchi et al. (13)	Tashiro et al. (14)	Miyauchi et al. (15)
Study Design	Observational cohort	Observational cohort	Prospective non-randomized
Sample Size	596 AF patients	132 AF patients	288 AF patients
Mean Age (years)	64.9 ± 10.0	62.2 ± 10.6	64.9 ± 10.0
Male Sex (%)	69%	72.7%	66%
AF Type (Paroxysmal, %)	61%	100% (paroxysmal only)	57%
Periodontitis Assessment	Serum IgG anti-P. gingivalis	Clinical periodontal exam (PPD ≥4 mm + BoP)	PISA
Follow-up Duration	17.1 ± 14.5 months	Median 3.0 (IQR: 1.1–6.4) years	507 ± 256 days
Outcomes	AF recurrence	Atrial arrhythmia recurrence (AF, AFL, AT)	AF recurrence
Key Findings	P. gingivalis type IV associated with AF recurrence	Periodontitis linked to higher arrhythmia recurrence	High PISA predicted AF recurrence

AF, atrial fibrillation; AFL, atrial flutter; AT, atrial tachycardia; PPD, probing pocket depth; BoP, bleeding on probing; PISA, periodontal inflamed surface area; HR, hazard ratio; IQR, interquartile range.

## Results

### Study selection

**Figure 1:**
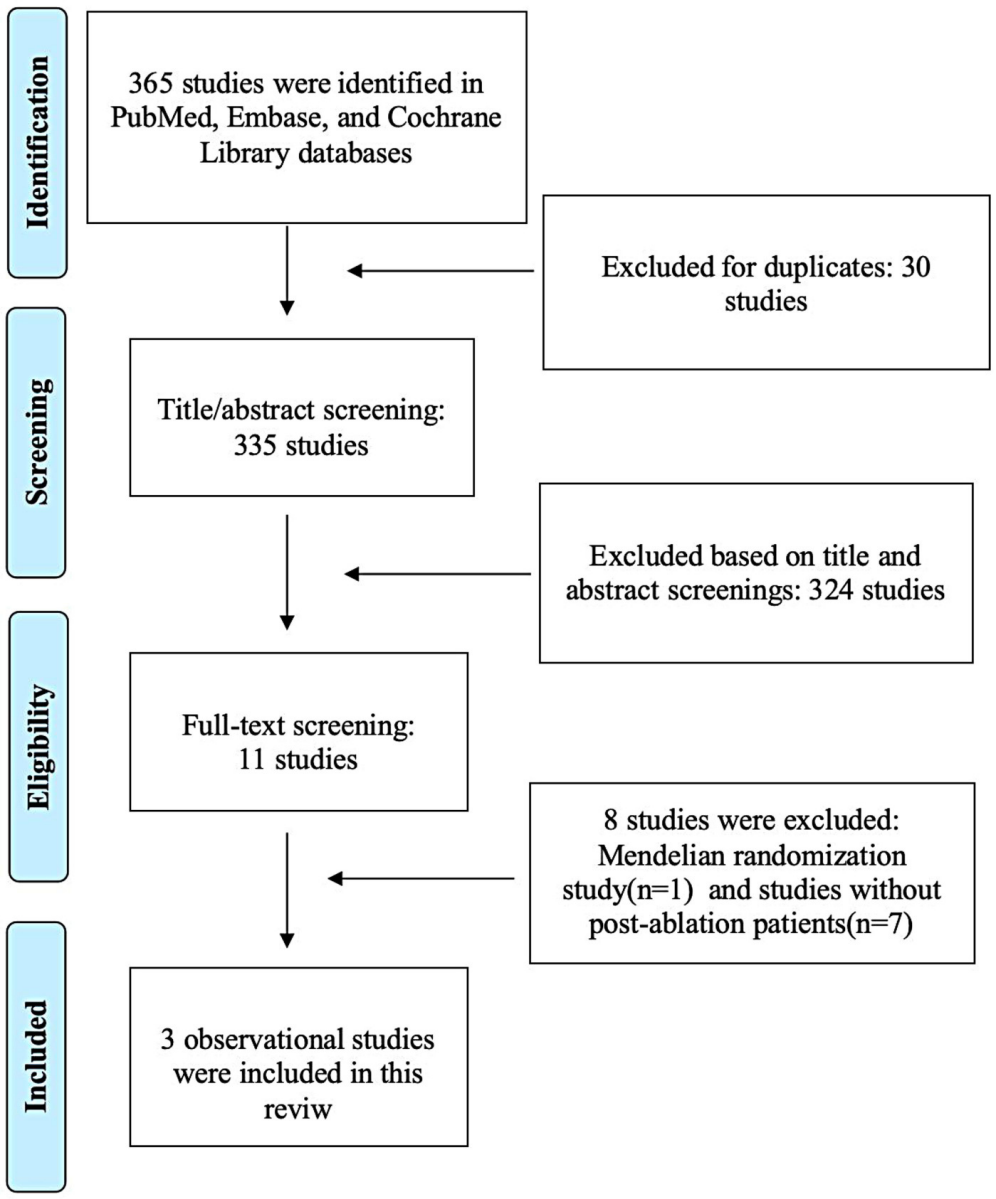
PRISMA flow diagram of study selection for the association between periodontitis and atrial fibrillation recurrence.

Figure 1 presents the PRISMA flow diagram depicting the study selection process. The initial search identified 365 records. After removing duplicates and screening titles and abstracts, 335 records were excluded. Eleven full-text articles were then assessed for eligibility. Of these, 8 studies were excluded due to methodological ineligibility: one employed a Mendelian randomization design (20), while the others did not specifically address AF recurrence in post-ablation patients (11, 12, 21–25). Ultimately, three observational studies (13–15) met all inclusion criteria and were included in this systematic review.

### Baseline characteristics of included studies

Baseline characteristics of the included studies are summarized in Table 1. The three included studies collectively enrolled 1,016 patients undergoing AF ablation, with individual sample sizes ranging from 132 to 596. The mean age ranged from 62.2 to 64.9 years, reflecting an older adult cohort typical of AF populations. Male predominance was consistent across studies (69%–72.7%). Common cardiovascular comorbidities included hypertension (49.2%–61%), diabetes mellitus (9.8%–20%), and dyslipidemia (30.3%–49.2%).

Periodontal status was assessed via differing methodologies: Miyauchi et al. (13) measured serum IgG antibody titers against Porphyromonas gingivalis subtypes; Tashiro et al. (14) employed clinical probing depth (≥4 mm with bleeding on probing); Miyauchi et al. (15) utilized the PISA index. The prevalence of periodontitis ranged from 54% to 57%. Echocardiographic parameters, particularly left atrial volume (68.4–78.7 ml) and diameter (37.3–40.5 mm), were elevated among patients with AF recurrence. Post-ablation antiarrhythmic drug use varied (22%–67%), while procedural success, particularly pulmonary vein isolation, was consistently high. These characteristics indicate a patient population with a substantial burden of cardiovascular and periodontal disease.

### Quality assessment

The methodological quality of the three included observational studies was assessed using the NOS tool, with all studies achieving scores ≥6. Miyauchi et al. (13) received a score of 7, demonstrating strengths in selection (e.g., a representative cohort and validated periodontal exposure based on serum Porphyromonas gingivalis antibody titers) and outcome assessment (standardized monitoring for AF recurrence). However, the study's comparability was limited by incomplete adjustment for relevant confounders, such as smoking status and oral hygiene practices. Tashiro et al. (14) achieved a score of 8, with detailed periodontal assessments by certified specialists and robust adjustment for age, sex, and CRP levels, although the duration of follow-up varied. Miyauchi et al. (15) also scored 7, supported by rigorous exposure measurement using the PISA index and multimodal methods for AF recurrence ascertainment. Nonetheless, potential residual confounding remained due to limited data on behavioral and socioeconomic factors. All studies adequately addressed attrition bias through structured and complete follow-up protocols, reinforcing the reliability of findings despite inherent limitations of observational study designs.

### Association of periodontitis with AF recurrence after ablation

Two studies (13, 14) evaluated periodontitis as an exposure, whereas one study primarily evaluated the association between periodontal treatment during the blanking period and AF recurrence (15). Given this methodological distinction, results from the observational study were interpreted separately and not pooled with exposure-based associations.

**Figure 2:**
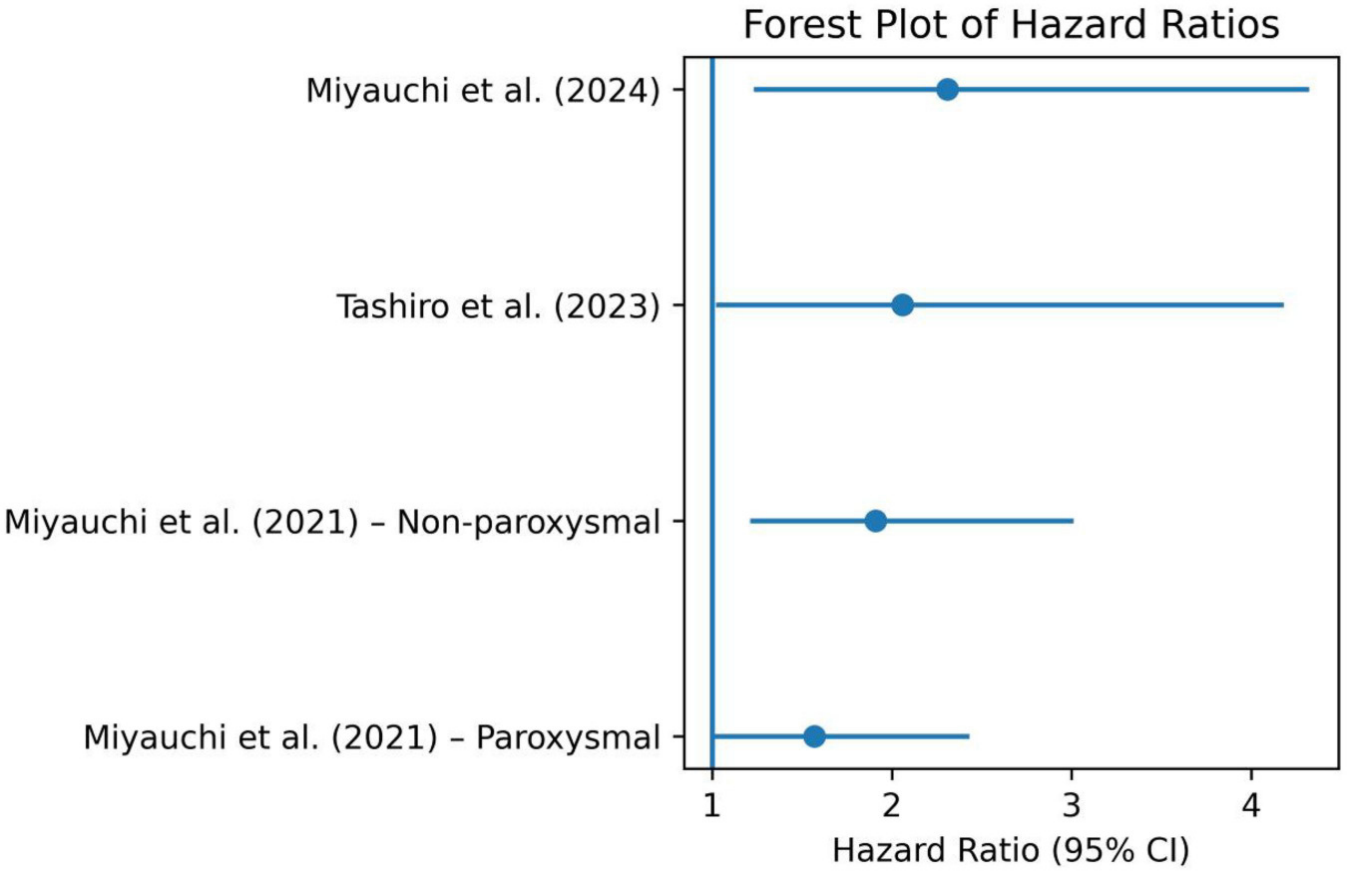
Forest plot of hazard ratios from the included studies.

Miyauchi et al. (13) observed that elevated serum IgG antibody titers against P. gingivalis type IV (a key periodontal pathogen) were independently associated with late AF recurrence (paroxysmal AF subgroup: HR = 1.57; 95% CI, 1.01–2.43; non-paroxysmal AF subgroup: HR = 1.91; 95% CI, 1.21–3.01) (Figure 2), particularly among patients with non-paroxysmal AF. Similarly, Tashiro et al. (14) found that clinically diagnosed periodontitis, defined by probing depth ≥4 mm with bleeding on probing, was associated with worse arrhythmia-free survival (HR 2.06, 95% CI 1.02–4.18) (Figure 2), with a stronger effect observed after the first post-ablation year.

The periodontal intervention in the recent study by Miyauchi et al. (15) was a standardized nonsurgical periodontal treatment protocol conducted during the 3-month blanking period following radiofrequency catheter ablation. Specifically, treatment was performed at 1 and 3 months post-ablation. It consisted of oral hygiene instruction followed by full-mouth debridement (both supragingival and subgingival) using an ultrasonic device and Gracey curettes, administered by an experienced dental clinician. After debridement, all tooth surfaces were polished with rubber cups and prophylaxis paste. The treatment did not require interruption of anticoagulation therapy, and no treatment-related complications were reported. This structured protocol ensured consistent delivery of care, allowing for clear clinical interpretation and reproducibility of the intervention. The authors found that high PISA values (>615 mm^2^) were predictive of AF recurrence (HR 2.31, 95% CI 1.23–4.32) (Figure 2). Notably, periodontal treatment during the blanking period was associated with a significant reduction in recurrence risk (HR 0.39, 95% CI 0.22–0.72), highlighting the therapeutic relevance of a modifiable oral inflammatory burden.

The underlying mechanisms proposed across studies converged on systemic inflammation and microbial-driven atrial remodeling. Miyauchi et al. (13) identified elevated inflammatory cytokines, including IL-6 and TNF-α, in patients with high P. gingivalis antibody titers. Miyauchi et al. (15) further reported increased levels of CRP and other interleukins in the high-PISA group. Tashiro et al. (14) added to this evidence, demonstrating elevated CRP levels (≥0.1 mg/dl) in patients with periodontitis. Additionally, P. gingivalis and Fusobacterium nucleatum were specifically implicated in recurrence among patients with non-paroxysmal AF (13, 15).

## Discussion

**Figure 3:**
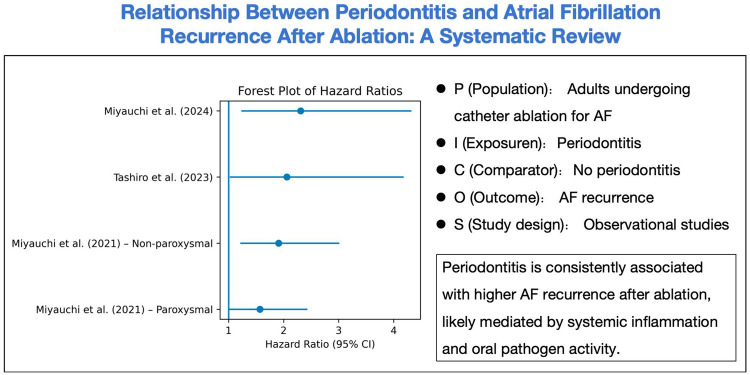
Relationship between periodontitis and atrial fibrillation recurrence after ablation.

The three included observational studies consistently found that coexisting periodontitis significantly increases the risk of AF recurrence after catheter ablation. Miyauchi et al. (13) reported that high serum IgG titers against the periodontal pathogen P. gingivalis were independently associated with late AF recurrence. Tashiro et al. (14) similarly observed that clinically diagnosed periodontitis predicted poorer arrhythmia-free survival. In aggregate, these observational findings align with broader epidemiologic data showing that periodontitis increases AF risk, and they identify periodontal inflammation as a potentially modifiable risk factor (26). Notably, the most recent study by Miyauchi et al. (15) demonstrated that treating periodontal disease during the post-ablation blanking period markedly improved outcomes: patients with a high PISA who received periodontal therapy had a ∼61% lower recurrence risk compared to untreated patients. Taken together, these results converge on the conclusion that oral inflammation and specific periodontal pathogens (especially P. gingivalis) are associated with worse ablation outcomes (Figure 3), suggesting an oral-cardiac axis in AF pathophysiology.

The link between periodontitis and AF recurrence likely reflects systemic inflammatory and microbial pathways. Chronic periodontal infection elicits persistent elevation of inflammatory mediators (e.g., CRP, IL-6, TNF-α) that can promote atrial substrate remodeling. For example, Miyauchi et al. (13) observed higher systemic IL-6 and TNF-α levels in patients with severe periodontal inflammation and AF recurrence. Moreover, periodontal bacteria themselves may translocate into the circulation and directly affect the myocardium. Studies have detected DNA or antibodies to P. gingivalis (and other oral pathogens) in cardiac tissue, implicating microbial invasion in atrial fibrosis and electrical remodeling (15, 27–30). These findings align with experimental models where periodontitis increased AF inducibility and inflammatory markers in animal studies (31). These observations are consistent with the concept of an oral-systemic inflammator*y* axis: periodontitis generates a low-grade inflammatory burden and pathogen-driven immune responses that predispose to atrial remodeling and arrhythmogenesis. Importantly, interventions that reduce periodontal inflammation (e.g. non-surgical periodontal therapy) have been shown in other contexts to lower systemic cytokine levels (32, 33), providing a plausible mechanism by which periodontal therapy could favorably impact AF outcomes.

The current findings dovetail with prior evidence linking poor oral health to AF. Large cohort studies (e.g., Atherosclerosis Risk in Communities Study) have found that severe periodontal disease predicts incident AF, and that regular dental care is associated with lower AF risk (22). A recent meta-analysis likewise reported that patients with periodontitis have a significantly higher risk of new-onset AF/flutter (26). Our systematic review adds to this evidence by focusing on the post-ablation setting: despite differences in study design and periodontitis assessment, all studies showed worse ablation outcomes in the presence of active periodontal disease. The consistency of the association across diverse populations reinforces its credibility. Furthermore, the demonstrable benefit of periodontal treatment after ablation (as reported by Miyauchi et al. 15), supports the idea that this risk is clinically actionable. Taken together, these data suggest that maintaining periodontal health may be a valuable adjunctive strategy in comprehensive AF care.

This systematic review highlights a consistent association between active periodontitis and increased AF recurrence after ablation. Periodontal inflammation and pathogens (notably P. gingivalis) emerged as strong risk factors in multiple studies, and preliminary interventional evidence suggests that addressing periodontal disease can significantly improve ablation success. These findings suggest that oral health should be integrated into AF management: for example, pre-ablation screening for periodontitis and timely dental care may help optimize arrhythmia outcomes. Future research should include well-designed prospective trials of periodontal therapy in AF patients, as well as mechanistic studies (e.g., atrial tissue analyses, serial biomarker tracking) to clarify causality. Ultimately, if these associations prove causal, they would support a multidisciplinary approach (linking cardiology and dentistry) to reduce AF recurrence and improve cardiovascular health. Future prospective studies incorporating standardized periodontal assessments, randomized periodontal interventions, and mechanistic investigations are warranted to establish causality and evaluate whether periodontal treatment can improve ablation outcomes.

The relevance of inflammation reduction in AF recurrence is further underscored by studies evaluating colchicine after AF ablation (34, 35). While some trials have demonstrated modest reductions in early post-ablation arrhythmia recurrence, others have reported neutral effects and limited tolerability, highlighting the complexity of systemic anti-inflammatory therapy. In this context, periodontal treatment represents a targeted strategy to reduce a chronic inflammatory source, potentially achieving sustained modulation of inflammatory burden with fewer systemic adverse effects.

## Limitations

Despite the coherence in observed associations, several limitations must be acknowledged. First, all included studies employed observational designs, which inherently preclude causal inference and are susceptible to residual confounding. Unmeasured variables, such as oral hygiene behaviors, dietary patterns, genetic susceptibility, and socioeconomic status, could influence both periodontal status and AF recurrence. Second, there was notable heterogeneity in the assessment of periodontitis, ranging from serologic antibody titers (13), to clinical probing criteria (14), to quantitative PISA (15). This variability may limit the comparability of findings and hinder the establishment of standardized thresholds for risk stratification. Third, definitions of AF recurrence varied across studies, including differences in monitoring techniques (e.g., intermittent Holter monitoring vs. continuous event recording) and the handling of the blanking period. Additionally, although systemic inflammation was consistently implicated as a mechanistic link, none of the included studies performed serial biomarker analyses, immune cell profiling, or histologic validation using atrial tissue samples to confirm causality. Finally, the generalizability of findings may be constrained by the single-center design of two studies and the predominance of older male patients in all three cohorts.

## Conclusion

Periodontitis is a plausible, modifiable risk factor for AF recurrence post-ablation, mediated by systemic inflammation and pathogen-specific effects. Integrating periodontal screening and treatment into AF care pathways may improve outcomes, but rigorous prospective trials are needed to confirm these observational findings. This review underscores the importance of interdisciplinary collaboration between cardiologists and dental specialists to optimize holistic AF management.

## Data Availability

The original contributions presented in the study are included in the article/Supplementary Material, further inquiries can be directed to the corresponding author.
